# Effects of electroacupuncture therapy for depression: Study protocol for a multicentered, randomized controlled trial

**DOI:** 10.1097/MD.0000000000022380

**Published:** 2020-09-18

**Authors:** Wa Cai, Wen Ma, Ai-Wen Chen, Wei-Dong Shen

**Affiliations:** a Institute of Acupuncture and Anesthesia; b Department of Acupuncture, Shanghai Shuguang Hospital Affiliated to Shanghai University of Traditional Chinese Medicine, Shanghai, China.

**Keywords:** electroacupuncture therapy, depression, protocol, multicentered, randomized controlled trial

## Abstract

**Introduction:**

As a major public health problem, depression has a negative impact on individuals and society. The aim of this well-designed trial is to evaluate the efficacy and safety of electroacupuncture (EA) treatment for depression.

**Methods/Design:**

A 3-arm parallel, nonblinded, randomized controlled trial will be performed in 4 hospitals (centers). A total of 144 participants will be divided into 3 groups: EA group, manual acupuncture (MA) group, and western medicine group. Participants in EA group and MA group will receive 12 sessions of acupuncture treatment for 4 weeks. Participants allocated to western medicine group will only take 20 mg fluoxetine orally per day for 4 weeks. The primary outcome is Hamilton Depression Scale. Secondary outcomes are Self-Rating Depression Scale, Depression Scale of traditional Chinese medicine (Depression Scale of Traditional Chinese Medicine), brain fMRI and blood biomarkers including neurotransmitters serotonin, dopamine, noradrenaline, inflammatory cytokines inerleukin (IL)-1β, tumor necrosis factor-α, IL-6, and neurotrophin BDNF. All the outcomes will be assessed at baseline, 4 weeks after EA treatment onset and 6-month follow-up.

**Discussion:**

The results of this trial will verify the efficacy and safety of EA treatment for depressive patients and provide acupuncturists and clinicians with robust clinical evidence.

**Trial registration:**

Chinese Clinical Trial Registry identifier: ChiCTR1900023420. Version 1. Registered on 26 May 2019. http://www.chictr.org.cn/edit.aspx?pid=37621&htm=4

## 1 Introduction

Characterized by emotional, behavioural, and cognitive features, symptoms of depression include dysphoric mood, feelings of hopelessness, loss of interest, social withdrawal, eating disorder, insomnia, fatigue, low self-esteem, and poor concentration. As a major public health problem, depression has a negative impact on individuals and society. A recent meta-analysis^[1]^ benchmarking the prevalence of depression in the community from 30 countries between 1994 and 2014 reported that the 1-year and lifetime prevalence of depression was 7.2% and 10.8%, respectively. Depression is associated with impaired physical or mental function^[2]^ and a substantial role loss in social life.^[3]^ It was found to have a worse impact on quality of life when compared with chronic diseases such as hypertension or diabetes.^[4]^ Furthermore, health services for depressed patients will bring about considerable financial costs.^[5]^

In most cases, depressed patients are treated with antidepressants^[6]^ such as citalopram, escitalopram, nortriptyline, milnacipran, mirtazapine, piracetam, and fluoxetine. Whereas, antidepressants have many side effects such as nausea, fatigue, insomnia, dry month, blurred vision, constipation, sexual problems.^[7]^ It was reported that Australians and Americans with depression had a preference for complementary therapies.^[8–10]^ Based on the theory of Traditional Chinese Medicine (TCM), acupuncture has a long history of use. Since that electroacupuncture (EA) was reported to effectively reduce depressive behaviors with few side effects,^[11]^ studies have found that EA can regulate neurotransmitter levels of serotonin (5-HT), noradrenaline (NE), and dopamine (DA) in animal models of depression.^[12]^ EA has been verified to trigger functional magnetic resonance imaging (fMRI) changes in the default mode network, anterior cingulate cortex, and amygdala-hippocampal formation^[13,14]^ where dysfunction has been previously detected in depressive disorders.^[15]^ Inflammatory cytokines including interleukin (IL)-1β, tumor necrosis factor (TNF)-α, and IL-6 were found to be changed in patients with depression treated by EA^[16]^ which was also capable of affecting neurotrophin levels of brain derived neurotrophic factor (BDNF) in depressed rats.^[17]^

However, a recent Cochrane meta-analysis^[18]^ focusing on the effectiveness of acupuncture or EA treatment for depression revealed that acupuncture or EA's effect compared with medication in reducing depression severity was unclear owing to the very low quality of evidence. Considering most trials did not report adverse events (AE), the risk of AEs with acupuncture or EA was also uncertain. Besides, few studies included follow-up assessment. Thus, a high-quality randomized controlled trial (RCT) is urgently needed. This protocol has been designed to conduct a multicentered, randomized controlled trial to assess the clinical efficacy and safety of EA for depression.

## 2 Methods/Design

### 2.1 Study design

**Figure 1:**
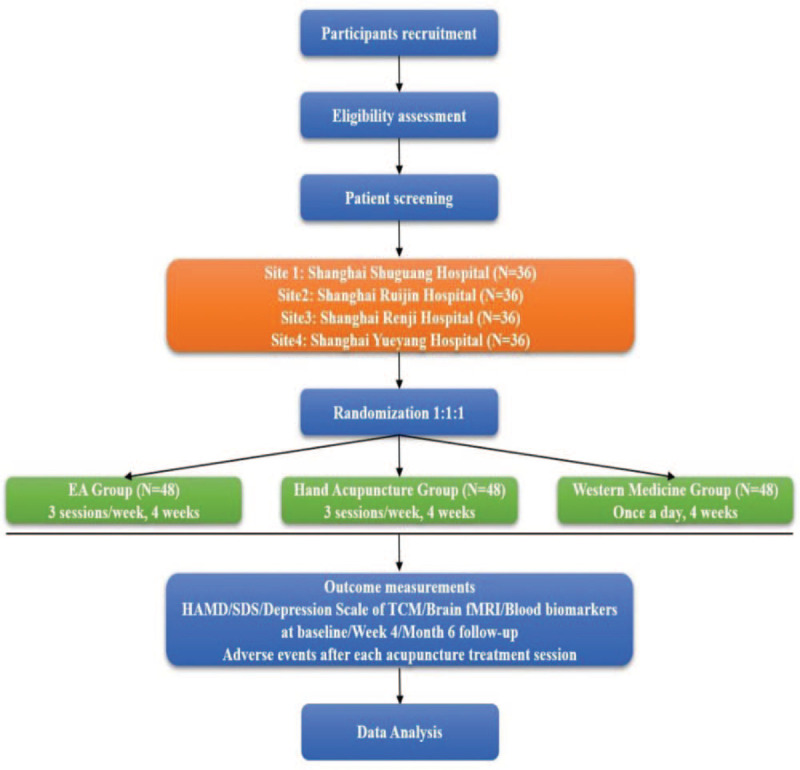
The planned flowchart of the trial.

**Figure 2:**
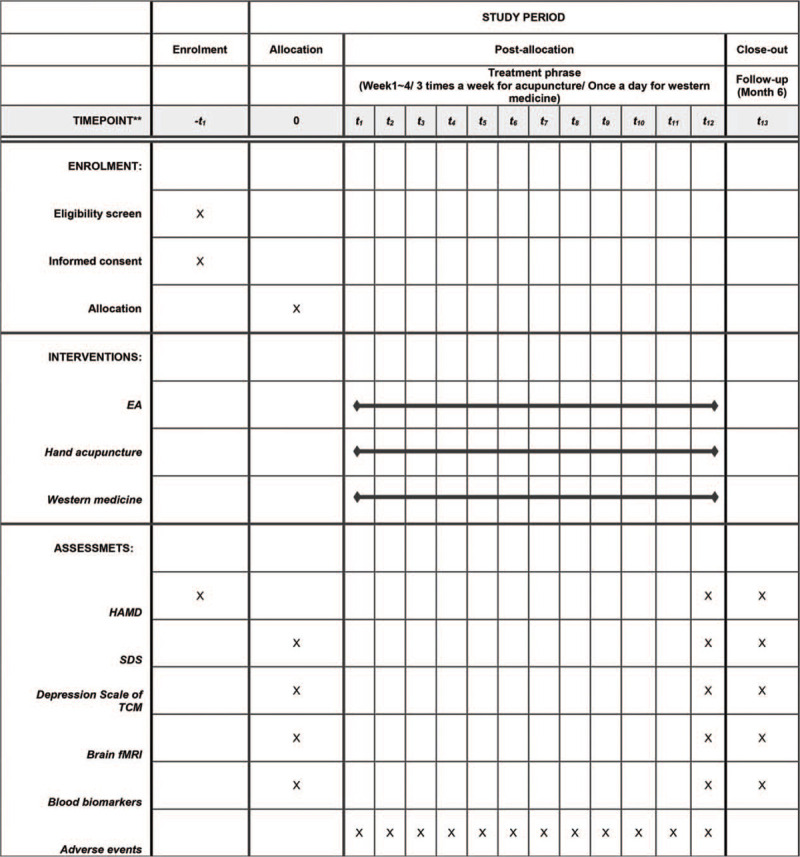
The study schedule for enrollment, treatments, outcome measurements and data collection. HAMD = Hamilton Depression Scale, SDS = Self-rating Depression Scale.

According with the Consolidated Standards of Reporting Trials^[19]^ and Standards for Reporting Interventions in Clinical Trials of Acupuncture guidelines for acupuncture studies,^[20]^ a multicentered, parallel, randomized controlled trial will be conducted from June 2019 to June 2021 (Fig. 1). Figure 2 indicates the study schedule for enrollment, treatments, outcome measurements, and data collection. The SPIRIT checklist can be found in Additional file 1.

### 2.2 Participants

#### 2.2.1 Patient screening

To conduct the safety assessment of patients and exclude patients with fatal conditions such as renal failure, heart failure, hepatic impairment, patient screening will be done by an independent researcher. It will depend on history inquiry, history recording, and laboratory tests which include complete blood count, urine analysis, fecal analysis, electrocardiograph, function of kidney, liver, and thyroid.

#### 2.2.2 Inclusion criteria

Patients who meet the following criteria will be enrolled in the trial: meet the diagnostic criteria of depression according to the Diagnostic and Statistical Manual of Mental Disorders, Fifth Edition^[21]^; aged 18∼75 years old, males or females; scores of Hamilton Depression Scale 17 (HAMD-17) are >17; able to understanding and answering the questions of scales; did not receive acupuncture treatment in the past month; willing to sign informed consents.

#### 2.2.3 Exclusion criteria

Patients who meet any of the following criteria will be excluded from the trial: undergoing other anti-depressive treatments within 1 month; history of personality disturbance, psychiatric disorders or brain diseases; complications of severe heart failure, infectious diseases or immune deficiency; having suicide thoughts; history of claustrophobia; having metal in their bodies; unable or unwilling to finish the whole trial.

#### 2.2.4 Elimination criteria

Data from patients who meet any of the following criteria after treatment will be eliminated from the analyses of this trial: occurrence of severe AEs; aggravation of depression severity; poor compliance.

#### 2.2.5 Recruitment strategies

Participant recruitment will take advantage of several strategies. Participants in this RCT will be mainly recruited from Shanghai Shuguang Hospital, Shanghai Ruijin Hospital, Shanghai Renji Hospital, and Shanghai Yueyang Hospital. Posters which will be put up in those 4 hospitals include brief introductions about the purpose, procedures, free treatments, possible risks of the trial, and researchers’ contact information. After screening test, another researcher will assign those participants who have signed the consent forms to different intervention groups. The recruitment process will be conducted by a clinical recruitment staff.

#### 2.2.6 Randomization and allocation

A computer program (Strategic Applications Software [SAS], version 9.1.3 [SAS Institute Inc., Cary, NC]) will be used by a statistician to generate the random allocation sequence. Four hospitals will be chosen as strata by using a stratified block randomization scheme. Opaque envelopes will be used to seal generated sequences and delivered to each center. Having enrolled participants meeting the selection criteria, the researcher will open the random allocation envelope in front of each participant one by one and assign him to 1 of 3 groups.

### 2.3 Blinding

This study will not be blinded.

### 2.4 Interventions

The enrolled participants will be allocated into 3 groups: electroacupuncture (EA) group, manual acupuncture (MA) group, or western medicine group.

Participants in EA group and MA group will receive 12 sessions of acupuncture treatment for 4 weeks, 3 times a week by experienced acupuncturists to ensure identical acupuncture treatment according to the standard operating procedures using stainless steel disposable needles (Energy, 40 mm in length and 0.25 mm in diameter, Jiajian Medical Supplies Co Ltd, Jiangsu, China). The same acupoints will be selected for participants in these 2 acupuncture groups. After skin sterilization, acupuncturists will puncture DU20, DU24, and GB8 horizontally 8 mm into the scalp skin. LR3, LI4, GB20, LI11, KI3, GB34, ST36 will be punctured vertically 15 mm into the skin, and only PC6 will be punctured 8 mm into the skin vertically to keep off the median forearm nerve. Needle manipulation including lifting, thrusting, and rotating will be done to obtain needling sensation (de-qi sensation). Acupoints of DU20, DU24, and bilateral ST36 will be stimulated using an electric current at a frequency of 2/15 Hz by EA device (Hwato, SDZ-II, Suzhou Medical Supplies Co Ltd, Jiangsu, China) in EA group, whereas EA device will not be used in MA group. The needle retention time for both two acupuncture groups will be 30 minutes for each session.

Participants allocated to western medicine group will only take 20 mg fluoxetine orally per day for 4 weeks instead of receiving acupuncture treatment.

### 2.5 Outcome

#### 2.5.1 Primary outcome

The primary outcome of the clinical study will be the score changes of Hamilton Depression Scale (HAMD) between baseline, 4 weeks after EA treatment onset, and 6-month follow-up.

##### 2.5.1.1 HAMD

The HAMD is a doctor-rated questionnaire designed to assess depression severity by rating multiple dimensions including probing mood, feelings of guilt, suicide ideation, insomnia, agitation or retardation, anxiety, weight loss, and somatic symptoms with a score on a 3 or 5 point scale.^[22]^ Scored by the researcher, the scale can present objective and reflective results. A score of 0 to 7 is considered to be “normal,” whereas scores of ≥20 indicate “moderate or severe depression.” The assessment time is 20 minutes.^[23]^

#### 2.5.2 Secondary outcomes

Secondary outcomes will be scored at the same time points as primary outcome, including Self-Rating Depression Scale (SDS), Depression Scale of traditional Chinese medicine (Depression Scale of TCM), brain fMRI, and blood biomarkers.

##### 2.5.2.1 SDS

Designed to assess depression severity for patients diagnosed with depression,^[24]^ the SDS is a short self-rated questionnaire consisting of 30 items that rate the affective, psychological, and somatic symptoms associated with depression. Assessed by the patient, the results of the scale are comparatively direct and subjective. Each item is scored on a scale of 1∼4. Depression severity is classified into “normal” (score 20∼44), “mild” (score 45∼59), “moderate” (score 60∼69), and “severe” (score ≥70).^[25]^

##### 2.5.2.2 Depression Scale of traditional Chinese medicine (TCM)

Based on the theory of TCM, the depression scale of TCM includes 6 items concerning depression-related symptoms such as chest distress, belching, palpitation, insomnia, fatigue, irritability, and weeping. Each item is scored on a 0∼3 scale. Higher summed scores indicate severer symptoms of depression from the perspective of TCM.

##### 2.5.2.3 Brain fMRI

A General Electric (GE) signa 1.5T echo speed superconducting MRI scanner will be used to perform the fMRI measurements. Functional images will be acquired with an echo-planar imaging (EPI) sequence. During the resting-state fMRI acquisition, each participant will be required not to think of anything and keep their eyes closed without falling asleep for 4.5 minutes’ fMRI scanning. Statistical Parametric Mapping (SPM12), Resting-State fMRI Data Analysis Toolkit (REST), and Data Processing Assistant for Resting-State fMRI (DPARSF) will be used to conduct the preprocessing of fMRI data. First, the remaining images will be corrected for different slice acquisition timing and head motion. Second, the images will be spatially normalized to the standard EPI template in SPM12 and resampled to a voxel size of 3 × 3 × 3 mm^3^.Third, the images will be smoothed with an isotropic Gaussian kernel (FWHW = 4 mm) and temporal band-pass filtered (0.01–0.08 Hz). Finally, the Automated Anatomical Labeling (AAL) atlas will be used to segment brain signals.^[26]^ One hundred sixteen brain regions and 144 volumes will be obtained after preprocessing.

##### 2.5.2.4 Blood biomarkers

Blood samples of participants will be collected and clot for at least 30 minutes at room temperature and centrifuged at 3000 rpm for 10 minutes at 4°C. Obtained serum samples will be stored at −80 °C until the day of enzyme-linked immunosorbent assay (ELISA) experiment. Serum 5-HT and BDNF will be measured using ELISA kits from R&D Systems (Minneapolis, MN). Serum NE and DA will be measured using ELISA kits from Weiao Co., Ltd. (Shanghai, China). Interleukin (IL)-6, TNF-α, and IL-1β levels in serum will be measured using ELISA kits from Xitang Co., Ltd. (Shanghai, China).

### 2.6 Safety assessment

During the whole treatment, participants safety will be assessed to avoid adverse events (AEs). AEs of acupuncture mainly include local bleeding or pain, local redness or bruising, itching, and dizziness, whereas fluoxetine will bring about a series of AEs such as nausea, upset stomach, constipation, headaches, insomnia. A researcher will record the time of occurrence, severity, progress and treatment of AEs at each visit. Once any severe AE occurs, both the principal investigator and the institutional review board (IRB) will be informed and take prompt actions simultaneously.

### 2.7 Sample size

The data statistician will be responsible for the calculation of the required sample size in each group. Based on previous studies, the cure effects of EA, MA and western medicine for depression were 15.7%, 17.4%, and 2.9%, respectively. Considering a significance level of 0.05 and a statistical power of 0.9, the calculated sample size in total is 120. Assuming about a 20% drop rate at a 1:1:1 allocation ratio, 144 participants will be recruited in this trial with 36 for each group.

### 2.8 Data collection, management, and monitoring

Baseline characteristic data will be collected, which consist of name, age, sex, employment, highest education level, diagnosis, typical symptoms, frequency of symptom occurrence, previous treatment, and family history. Outcomes will be assessed at baseline, 4 weeks after EA treatment onset, and 6-month follow-up. In each research center, original forms including case report forms and Adverse Events Form will be carefully checked and input into the computer. The trial will be monthly monitored by the Data and Safety Monitoring Board (DSMB) of 4 centers. The data manager of the DSMB from each center has the right to access the final data set and approve others’ requests of accessing the final data set.

### 2.9 Statistical analysis

SPSS 24.0 (SPSS Inc., Chicago, IL) will be used to conduct the whole data analysis. The intention-to-treat (ITT) analysis will include any participant who has at least one treatment session. The “last observation carried forward” rule will be applied to manage missing data in ITT analysis of secondary outcomes, whereas the maximum likelihood estimation method will be used for primary outcome. Any participant who receives at least 9 treatment sessions during the trial will be included in per protocol analysis.

Considering the homogeneity of the baseline characteristics between groups, group *t* test for quantitative data and Chi test for qualitative data will be applied. Group *t* test for normally distributed data or Mann-Whitney *U* test for non-normally distributed data will be used to compare variables between groups. For differences within each group, repeated measured analysis of variance will be used to analyze assessments of different time points, whereas Wilcoxon signed-rank test will be selected for non-normally distributed data. A *P* value <.05 will be considered statistically significant (2-sided).

### 2.10 Quality control

To ensure the quality of this trial, supervisors from clinical evaluation center of Shanghai Shuguang Hospital will be responsible for the monitoring of case report forms, participants’ compliance with EA treatments, trial master file, serious AEs records, and data set regularly and strictly based on the quality standard of good clinical practice.

### 2.11 Clinical trial registration

This RCT has been applied for registration in the Chinese Clinical Trial Registry (ChiCTR1900023420, http://www.chictr.org.cn/edit.aspx?pid=37621&htm=4).

## 3 Discussion

EA has been found to be effective in treating with depression with few side effects in a number of clinical studies,^[27–30]^ but an updated Cochrane review^[18]^ concluded that there was lack of solid evidence to recommend that EA is a beneficial treatment for depressive patients in many aspects due to poor quality of previous trials, which required further high-quality trials to support the recommendation. Besides, fluoxetine, as a selective serotonin reuptake inhibitor, is a widely used antidepressant. It was found to be effective in treating depression.^[31]^ This RCT is designed to compare the difference results of intervention by EA, MA, and fluoxetine to evaluate the efficacy of EA in treating depression and provide reliable clinical evidence.

The strengths of this RCT are associated with its multicentered design, concealed randomization, scales of multiple dimensions, imageological changes, specific biomarkers to better evaluating EA's efficacy. Based on the theory of TCM, the combination of acupoints DU20, DU24, GB8, LR3, LI4, GB20, LI11, KI3, GB34, ST36, and PC6, most frequently used for treating depression, can alleviate depressive symptoms by dispersing liver qi and relieving qi stagnation.

However, limitations in this trial should also be noted. It is unfeasible to blind the acupuncturists or clinicians who will treat participants from three groups differently. Meanwhile, participants allocated to different groups will definitely accept different treatments. Thus, participants cannot be blinded either. Double blinding is hard to be performed in this RCT.

As a conclusion, according to the CONSORT^[19]^ and STRICTA^[20]^ guidelines, the aim of this trial is to provide acupuncturists and clinicians with robust evidence of the efficacy and safety of EA treatment for depressive patients.

## Author contributions

The trial was designed by MW, CAW and CW. CW drafted the manuscript, which was carefully revised and discussed by MW and SWD. All authors read and approved the final manuscript.

**Conceptualization:** Wa Cai, Wen Ma, Ai-Wen Chen, Wei-Dong Shen.

**Writing – original draft:** Wa Cai.

## Abbreviations

Abbreviations: 5-HT = serotonin, AE = adverse event, CBC = complete blood count, DA = dopamine, DSMB = Data and Safety Monitoring Board, EA = electroacupuncture, ECG = electrocardiograph, HAMD = Hamilton Depression Scale, IRB = institutional review board, ITT = intention-to-treat trials, MA = manual acupuncture, NE = noradrenaline, RCT = randomized controlled trial, SDS = Self-Rating Depression Scale.
